# Reminiscences of Half a Century of Life in the World of Theoretical Physics

**DOI:** 10.3390/e26020158

**Published:** 2024-02-11

**Authors:** Constantino Tsallis

**Affiliations:** 1 Centro Brasileiro de Pesquisas Físicas and National Institute of Science and Technology of Complex Systems, Rua Xavier Sigaud 150, Rio de Janeiro 22290-180, RJ, Brazil; tsallis@cbpf.br; 2 Santa Fe Institute, 1399 Hyde Park Road, Santa Fe, NM 87501, USA; 3 Complexity Science Hub Vienna, Josefstädter Strasse 39, 1080 Vienna, Austria; 4 Sistemi Complessi per le Scienze Fisiche, Socio-Economiche e della Vita, Dipartimento di Fisica e Astronomia Ettore Majorana, Università degli Studi di Catania, Via S. Sofia 64, 95123 Catania, Italy

**Keywords:** critical phenomena, graph theory, nonlinear dynamical systems, nonadditive entropies, nonextensive statistical mechanics

## Abstract

Selma Lagerlöf said that culture is what remains when one has forgotten everything we had learned. Without any warranty, through ongoing research tasks, that I will ever attain this high level of wisdom, I simply share here reminiscences that have played, during my life, an important role in my incursions in science, mainly in theoretical physics. I end by presenting some perspectives for future developments.

***Yo soy yo y mi circunstancia***

[José Ortega y Gasset]

## 1. The Leitmotif

Let me start this narrative backwards. While trying to organize a plethora of reminiscences from my scientific life, a single aspect vividly came to my mind that percolates through it all: the intuitive or conscious search for beauty, either primordial or actual. Insistent thoughts and feelings such as “This way it is not well expressed”, “It must be possible to present it, to think of it, in a more powerful, more general form”, “Yes, now it is perfect, there is no way to present it or to think about it more beautifully or more simply” have been recurrent along my entire life, the *leitmotif* of my research activities. It is, almost always, through this path that I imagine—sometimes successfully—the correct scenario, the correct outcome, for a given issue. I have no doubts that such experiences are currently shared with virtually all scientists and artists. They are, in any case, shared with A. K. Rajagopal, with whom I lengthily discussed, many years ago, precisely this point.

The earliest memory that I have of some sort of conscious search for aesthetics goes back to my childhood. Every day, I used to commute between my residence and school in Mendoza, Argentina, using the tramway. Every tram ticket had a unique five-digit number. I had immense pleasure in utilizing every travel duration to play with the digits, rearranging them or performing various simple arithmetic operations so that the number would finally be written in a more beautiful manner. I would not stop until the number reappeared, in my child mind, in an *aesthetic* form.

For Plato, *Truth* and *Beauty* were two inseparable aspects of the same reality.

John Keats wrote, in 1819,

*Beauty is truth, truth is beauty*
*that is all Ye know on earth, and all Ye need to know.*

A few years later, Emily Dickinson, in her lonely style, insisted

*I died for beauty, but was scarce*
*Adjusted in the tomb,*
*In an adjoining room.*
*He questioned softly why I failed?*
*“For beauty”, I replied.*
*“And I for truth,—the two are one;*
*We brethren are”, he said.*
*And so, as kinsmen met at night,*
*We talked between the rooms,*
*Until the moss had reached our lips,*
*And covered up our names.*

**Figure 1:**
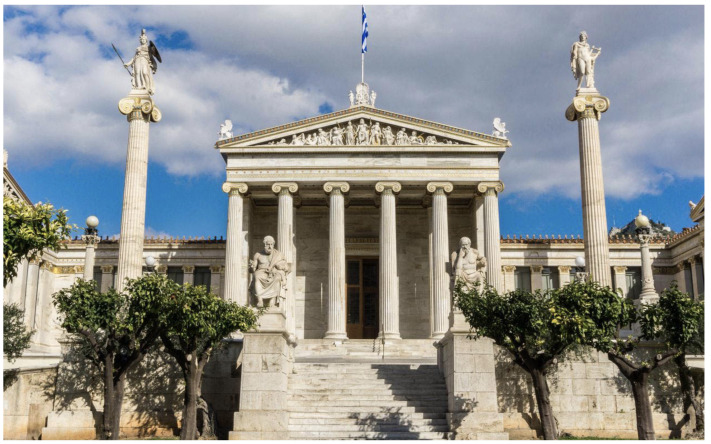
The Academy of Athens. Centered between the two columns of Pallas Athena and Apollo, we see the statues of Socrates and of his disciple Plato, the founder of the original Academy, where Aristotle studied for twenty years.

The *Academy of Athens* (in Greece, just *Academy*) is not an Academy of Sciences or of Arts, it is just *The Academy*, originally founded by Plato. And, in front of it, two big columns stand up, dedicated, one of them, to Pallas Athena, the Goddess of Wisdom and Science—the utopia of which is Truth, and the other one to Apollo, the God of Art—the utopia of which is Beauty—(see Figure 1).

**Figure 2:**
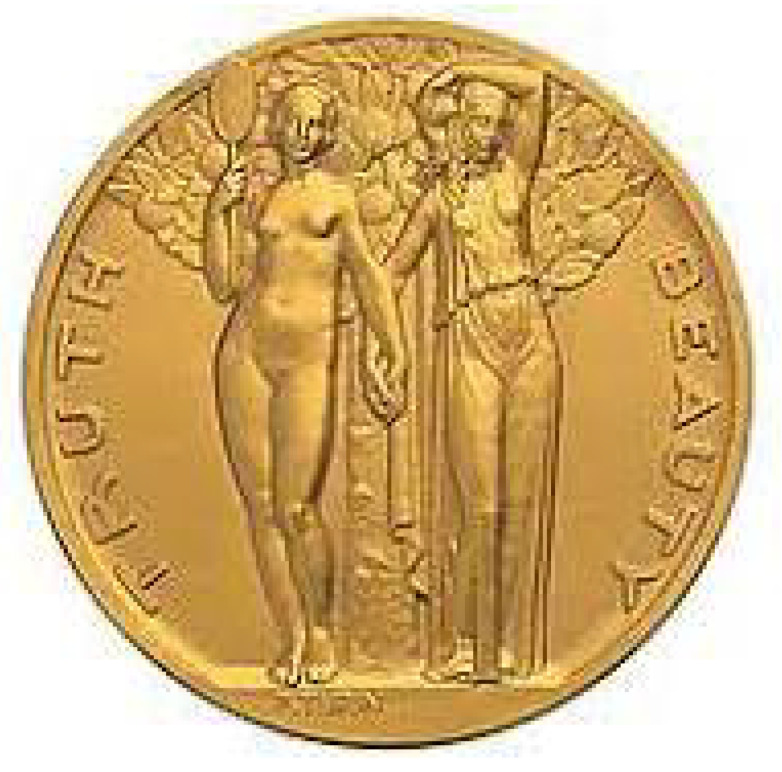
Symbol of the Institute for Advanced Study, in Princeton, New Jersey.

A few months ago, while lecturing in Princeton University, I had a pleasant surprise: the symbol of the celebrated Institute for Advanced Study where Einstein spent many years of his life precisely joins, hand in hand, Truth and Beauty (see Figure 2).

## 2. Early Years

After finishing my high-school studies, I first registered, at the National University of Cuyo, in San Juan, Argentina, on the course of Chemical Engineering at the (immature) age of 15 and, at the age of 16, I started to follow with enthusiasm the lessons in mathematics, physics and chemistry. During the first two years, the obligatory disciplines were the basic ones and I was enchanted. But, in the third year, the technological disciplines themselves started with all their weight and my interest definitively declined. Coincidentally, I heard a presentation by Alberto P. Maiztegui at the Institute of Physics Balseiro, part of the same University but located in the beautiful Bariloche. Fellowships were accessible through a national competition in Buenos Aires, one of the requirements being to have completely finished two full years of studies in exact or technological sciences. This is how I shifted from engineering to physics. I finished in 1965 and eventually moved to France, supported by a French fellowship, to take a doctorate degree (Doctorat d’ État ès Sciences Physiques).

After some initial years of research in experimental physics (the construction, in 1968, at the Laboratoire de Spectroscopie Moléculaire headed by Gilbert Amat at the University of Paris in Jussieu, of a molecular laser working on the CO and $CO_{2}$ states, and, in 1969, impedance measurements of complex perovskites supervised by Claude Rocchiccioli-Deltcheff [1] at the Laboratoire de Magnétisme et Physique des Solides/Centre National de la Recherche Scientifique in Bellevue-Paris), I definitively turned the focus of my research onto theoretical physics. However, that early experience in experimental physics indelibly marked my entire understanding of science.

My first theoretical steps were taken, in the area of ferroelectricity in perovskites [2,3], with Jacques Bouillot and Roland Machet, who were starting to work at the University of Dijon, France. We three together discovered with perplexity the beauty and power of the sum rules in physics.

Those years were marked by the May 1968 student generalized movements in Paris and elsewhere. I moved to the Service de Physique du Solide et Resonance Magnétique—Commissariat à l’Énergie Atomique, in Saclay-Paris (Jacques Villain had just moved from Saclay to Grenoble and I inherited his office room). There, by meditating on the fascinating mathematical and physical mysteries of phase transitions and critical phenomena, I had the invaluable fortune to learn priceless insights from Nino Boccara and Gobalakichena Sarma.

I still remember today that one of my first personal theoretical efforts had a definitively aesthetic motivation. K. Kobayashi [4,5] had proposed an interesting model for KDP-like ferroelectric crystals. There was, however, an issue of his theory that strongly bothered me: the order parameter and the frequency of the associated soft mode did *not* vanish at the same temperature. Since, within such simple theoretical approaches, I considered the order parameter and the corresponding soft mode to be two faces of the same coin, that discrepancy appeared to me as inadmissible. I thought that the simplest and most beautiful scenario was that the two main “consequences” of the same “cause”—the thermodynamical inclination of the system to make a phase transition—emerged together, at a *single* temperature. Then, by including some specific configuration energy within Kobayashi’s theory, it came out that, as desired, the order parameter and the soft mode frequency indeed vanish at precisely the *same* temperature. Satisfied with this result, I then published this, so corrected, theory [6]. The 1972 paper’s Figure 1 exhibits the critical temperature as a function of a scaled molecular field Γ. This particular dependence was, some time later, experimentally confirmed at the Institute of Physics of the University of Campinas-Unicamp. Unfortunately, I have not succeeded in finding the corresponding plot but I still remember Sergio Porto showing it to me at the University of Brasilia while, smiling, he told me “You propose and Unicamp checks!” That was my first experience where some theoretical effort of mine was experimentally validated. I was fascinated by this experimental verification which, for me, was close to a miracle. Beauty was showing its power in science…

This fact was somehow consistent with the two most influential lessons—one from Guido Beck (distinguished German physicist, former assistant of Werner Heisenberg, having lived part of his life in Argentina and Brazil, where he currently taught the theory of relativity and quantum mechanics; his quantum mechanics first lesson was simply unforgettable: he entered the classroom and, to our enormous perplexity, said abruptly “Do you think that an electron is a hard black little ball? Noooo, an electron is a distribution of amplitudes of probabilities!”), at the time I was his student at the Balseiro Institute of Physics in Bariloche, Argentina, and the other one from Pierre-Gilles de Gennes (French physicist, 1991 Nobel laureate in Physics), when I was following, at Orsay-Paris, his regular course on Solid State Physics—that I was fortunate to receive during my student times, before my initiation as a researcher.

Guido Beck was teaching us some simple features about the real and imaginary parts of some basic dissipative coefficient, and, in the middle of long mathematical calculations, a zero emerged. At this point he said “Because of this zero, if you put your finger into shoe polish, when you get your finger out, *the hole remains*”. Indeed, if you put your finger into water, the hole then disappears! That was like a sudden flash of lightening in my mind: *the good mathematical theory ought to reflect the empirical fact!* The protagonists of Raffaello Sanzio’s *School of Athens* in the Vatican are Plato and Aristotle. Plato point to the heavens (*topos Uranos*), in contrast to Aristotle who points towards the ground (*topos Physis*). In their search for truth, they were both right: the theoretical truth must correspond to the empirical truth, two faces of the same coin! The perfect balance between Poetry and History. From Aristotle’s thoughts: *Poetry is more philosophical and more elevated than history; for poetry expresses the universal, and history only the particular. History tells us the events as they happened, whereas poetry tells them as they could or should have happened*. (“Elevated” is to be understood here as closer to “philosophy”, which occupies, in the Aristotelian thinking, the highest place in the hierarchy of the forms of knowledge.)

In a different realm, in his teaching, Pierre-Gilles de Gennes solved concrete physical problems by using generic intuitive and scaling arguments. So, in a few minutes, he would find the correct answer (excepting perhaps for a pure number of the order of unity). At home, after many-hour calculations, we students verified systematically that his answer indeed was the correct one! This opened in my mind a completely new perspective: *It is possible to find the correct theoretical description and understanding without doing tedious mathematical calculations, just by focusing on what is a must, or nearly so, for that specific physical problem!* (It is within this respect that *abduction*, the Charles Sanders Peirce favorite form of logical inference, plays its central role, the one which enables Sherlock Holmes to identify the murderer, through the “relevant details”!).

Bond percolation is an interesting geometrical critical phenomenon based on *independent bond-occupancy probabilities* on a given structure, e.g., a square lattice. If we have a *series* array of two bonds whose occupancy probabilities are $p_{1}$ and $p_{2}$, the overall occupancy probability is given by

(1) $$p_{s}=p_{1}p_{2}\;,$$

where *s* stands for *series*. If the array is a *parallel* one, then the overall occupancy probability is given by

(2) $$p_{p}=p_{1}p_{2}+p_{1}\left(1-p_{2}\right)+p_{2}\left(1-p_{1}\right)=p_{1}+p_{2}-p_{1}p_{2}\;,$$

where *p* stands for *parallel*. But, already at this elementary stage, aesthetics may come in! We may say that Equation (2) is *not* written in a beautiful manner. Indeed, it can be rewritten in a much more elegant way, namely

(3) $$1-p_{p}=\left(1-p_{1}\right)\left(1-p_{2}\right)\;.$$

Now, all variables are written in *one and the same form*. On top of this, Equation (3) trivially transforms the parallel composition into the *same form* as the series one. This leads us naturally to a deep transformation which we will name *duality*, i.e., p↔(1−p). It is from these very elementary seeds that a powerful graph calculation algorithm, currently referred to in the literature as the *Break-Collapse Method*, was developed, valid for a model, namely the $q_{P}$-state Potts model (where *P* stands here for *Potts*). This model is sensibly more general than bond percolation, which is therein recovered as the particular case $q_{P}=1$. A very elegant method was born and an important unification was achieved based on the above very simple considerations [7,8]. Incidentally, a very useful variable currently referred to as *thermal transmissivity* *t* was concomitantly introduced (it was Robin B. Stinchcombe, during a car ride in Rio de Janeiro, who helped me, with his beautiful Oxford English, to decide whether to call it *transmissivity* or *transmittivity*); this variable precisely becomes the above bond occupancy probability *p* when $q_{P}=1$ (I once had an unforgettable conversation with my friend and outstanding statistical mechanicist Antonio Coniglio: with his *red bonds*, he had arrived at essentially the same understanding of this beautiful geometrical–thermal problem). The square lattice is a self-dual (infinite) graph in the sense that, if we cut each of its bonds by one and only one dual bond, we recover once again the square lattice. Because of this crucial topological property, its bond-percolation critical point $p_{c}$ must satisfy $p_{c}=1-p_{c}$; hence, $p_{c}=\frac{1}{2}$ (see [9,10]). Along totally analogous lines, it can be shown that the critical thermal transmissivity $t_{c}$ of the square-lattice $q_{P}$-state Potts ferromagnet is given by $t_{c}=\frac{1}{1+\sqrt{q_{P}}}$. I then introduced a convenient new variable, namely

(4) $$s\equiv \frac{\ln[1+\left(q_{P}-1\right)t]}{\ln q_{P}}$$

It is straightforward to verify that the duality (i.e., series–parallel) transformation now becomes s↔(1−s), *one and the same for all values of $q_{P}$*! Consequently, the square-lattice critical point is given by $s_{c}=\frac{1}{2}\;\left(\forall q_{P}\right)$ and the $q_{P}$-state Potts ferromagnetic model becomes, in this sense, collapsed in the bond percolation model, *for all values of $q_{P}$*. I believe that this simplicity illustrates well what makes the beauty of unification!

Notice, by the way, that definition (4) satisfies, in the $q_{P}\to 1$ limit,

(5) $$s∼\frac{\ln[1+\left(q_{P}-1\right)t]}{q_{P}-1}∼t\;.$$

Amazingly enough, we shall later on see [11] that this transformation is precisely the one which, through $\left(q_{P}-1\right)↔\left(1-q\right)$, connects the Rényi entropic functional $S_{q}^{R}$ (*R* standing for *Rényi*) and the nonadditive entropic functional $S_{q}$, which will play a major role in generalizing the Boltzmann–Gibbs statistical mechanics; *s* plays here the role of $S_{q}^{R}$ and *t* that of $S_{q}$, the $q_{P}\to 1$ (hence q→1) limit corresponding to the Boltzmann–Gibbs entropic functional $S_{BG}$ (Per Bak, the “father” of the concept of *self-organized criticality*, once shared with me a curious statement, “Every man has only one idea in his life; if he has many, he has none”. Many years later, at the Santa Fe Institute, NM, the Nobel laureate Murray Gell-Mann and I lengthily discussed, just for pleasure, this point: he disagreed with Bak’s statement, whereas I was inclined to agree with it).

Diverse real-space renormalization groups and other theoretical techniques were implemented on the Potts-model above grounds. This perspective enabled many doctoral theses to be worked out as well as several papers to be published. Also, basically due to these developments in theoretical physics, I had in 1982 the good fortune to become a Fellow of the John Simon Guggenheim Foundation (USA), which allowed me to have enriching post doc periods at Oxford, Boston and Cornell Universities.

The unification implied in any generalization always involves some form of simple beauty. Indeed, within a generalization, diverse physical situations emerge as particular instances of a more powerful, more “universal”, theory, a sort of metaphor.

It is a memory of this kind which points to the calculation that Anibal Omar Caride and myself performed in 1984 [12]. It concerned the quantum specific heat of an anisotropic rigid rotor whose inertial tensor has a revolution symmetry. This approach unified three different symmetries, namely the spherical, oblate (“flying disk” like) and prolate (“cigar” like) ones. The quantum nature of the problem ensures that the various rotational degrees of freedom are activated at possibly different temperatures *T*. In the extreme prolate case, rotations around the symmetry axis might be frozen until very high temperatures are achieved, due to its nearly vanishing moment of inertia *I*. Indeed, in such a case, the first excited state of rotation around that axis becomes thermally activated only at extremely high temperatures. This yields a nonuniform convergence related to the *ordering* of T→∞ and I→0. This fact elegantly clarifies the perplexity felt by Josiah Willard Gibbs concerning the specific heat of diatomic molecules calculated, naturally in his time, on *classical* grounds: quantum mechanics did not even exist! To be more precise, in the Preface of his celebrated 1902 book [13], he writes: *Even if we confine our attention to the phenomena distinctively thermodynamic, we do not escape difficulties in as simple a matter as the number of degrees of freedom of a diatomic gas. It is well known that while theory would assign to the gas six degrees of freedom per molecule, in our experiments on specific heat we cannot account for more than five. Certainly, one is building on an insecure foundation, who rests his work on hypotheses concerning the constitution of matter. Difficulties of this kind have deterred the author from attempting to explain the mysteries of nature…*

Another memory along similar lines refers to the 1987 discussion by Maria da Conceição de Sousa Vieira and myself focusing on the thermal equilibrium of a *D*-dimensional ideal gas in Gentile parastatistics [14]. Each parastatistics is characterized by the *maximal allowed number* $p_{G}$ of particles per state (*G* stands for *Gentile*); hence, $p_{G}=1$ and $p_{G}\to \infty$, respectively, recover Fermi–Dirac (FD) and Bose–Einstein (BE) statistics; in the BE case, a finite-temperature macroscopic condensation on the ground state occurs at a sufficiently high dimension *D*, whereas no such phenomenon is possible for the FD case. The central issue of that paper concerns whether such macroscopic condensation is or is not possible for $1<p_{G}<\infty$. The answer is that *it is not*. This strong result is once again related to an elegant nonuniform convergence, this time involving the ordering of the $p_{G}\to \infty$ and the chemical potential μ→0 limits. An analysis of this result on aesthetical grounds is available in [15].

## 3. Nonadditive-Entropy Years

As a consequence of the French–Brazilian *Colloquium on Phase Transitions (Critical Phenomena)* that I organized in November 1981 in Rio de Janeiro, a French–Mexican–Brazilian similar event, the *First Workshop on Statistical Mechanics*, was held in September 1985 in Mexico City. France provided important financial support. The French delegation was led by Édouard Brézin and the Brazilian one by myself. During a morning coffee break, while the participants were outside chatting, I remained resting inside the lecture room. Brézin was at the blackboard with a Mexican student whose name I do not remember. Their conversation was about something related to multifractals. I could not hear them because of the distance, but I could see that Brézin was writing on the blackboard various expressions containing $p^{q}$, well known to naturally appear in the theory of multifractals. Then, it suddenly came to my mind that it would be possible with $p^{q}$ to generalize Boltzmann entropy and therefore the entire standard statistical mechanics. Back home, I simply wrote down the expression of the following entropic functional:

(6) $$S_{q}=k\frac{1-\sum_{i=1}^{W}p_{i}^{q}}{q-1}\;\;\;\left(q\in \left[-\infty ,\infty \right]\right)\;.$$

If this expression, which trivially contains the Bolzmann–Gibbs–von Neumann–Shannon functional $S_{BG}=-k\sum_{i=1}^{W}p_{i}\ln p_{i}$ as the q→1 instance, was adopted as a postulate, then *it would be possible to generalize the entire BG statistical mechanics* [13,16,17,18,19]! (Years later, I gradually learnt from Silvio R.A. Salinas, Richard N. Silver and others that similar entropic functionals had previously been advanced in the literature of cybernetics and related mathematical formalisms. But, seemingly, no one ever addressed the possibility of generalizing, on such grounds, the entire BG theory itself. Historical details can be found in [20] and references therein.) During three years I did not feel like publishing anything along this line because I had no clarity about what could be the physical interpretation of $S_{q}$. Obviously, ideas related to hierarchical space–time structures were lurking. Also, for whatever reason, the thought emerged insistently that a small probability $p_{i}$ ($0<p_{i}\ll 1$) may be considerably magnified through the *biased* probability $p_{i}^{q}$ (more precisely, $p_{i}^{q}/\sum_{j=1}^{W}p_{j}^{q}$) if q<1, i.e., basically, ${\left(0.01\right)}^{1/2}=0.1\gg 0.01$. The image that appeared in my mind was the calm and gigantic vortices that I had seen in the bottom of the river at the fascinatingly hectic Iguaçu Falls: zillions of water molecules slowly turning around, just one after the other. An astronomically low a priori probability, stable and peaceful, quasi-stationary state in the middle of Hesiodic Chaos! (In fact, many years later, various connections of turbulent systems did appear with theoretical approaches involving q≠1, e.g., [21,22,23]).

Time goes by and, in 1987, Enaldo F. Sarmento, myself and a few other colleagues met, for a few days (*Encontro de Trabalho sobre Autômatas Celulares*, 24 to 28 August 1987, Maceió, Alagoas), at the Federal University of Alagoas in Maceió with the purpose of launching in Brazil the area of cellular automata. During a free-time period, on a blackboard, I presented the entropy $S_{q}$ to Hans J. Herrmann and Evaldo M. F. Curado, and we searched, without particular success, for possible physical applications. Next day, there were too many mosquitoes in the hotel where I was sleeping and I decided to go back to Rio de Janeiro. During the air flight I optimized the functional $S_{q}$ with the standard norm and energy constraints, and I found the now well-known *q*-exponential distribution. I was delighted by the fact that this distribution unified exponential, asymptotic power-law and cut-off behaviors, depending on whether q=1, q>1 or q<1.

Some time later on, virtually all the best statistical physicists of Brazil happened to be in my office at CBPF to discuss the organizational issues of the upcoming IUPAP Statphys 17 meeting to be held in 1989 in Rio de Janeiro. At the end of our discussion, I briefly presented to them, on the blackboard, the main lines of the $S_{q}$ proposal, and asked them whether it would be worthy or not to make this proposal in a standard international journal. The unanimous opinion was favorable to submitting it outside Brazil. I still remember the words of Silvio R. A. Salinas, at the time Chief Editor of the Brazilian Journal of Physics (BJP): “I would send it to a good journal outside Brazil, but if you want to submit it to BJP, it is already accepted!”.

**Figure 3:**
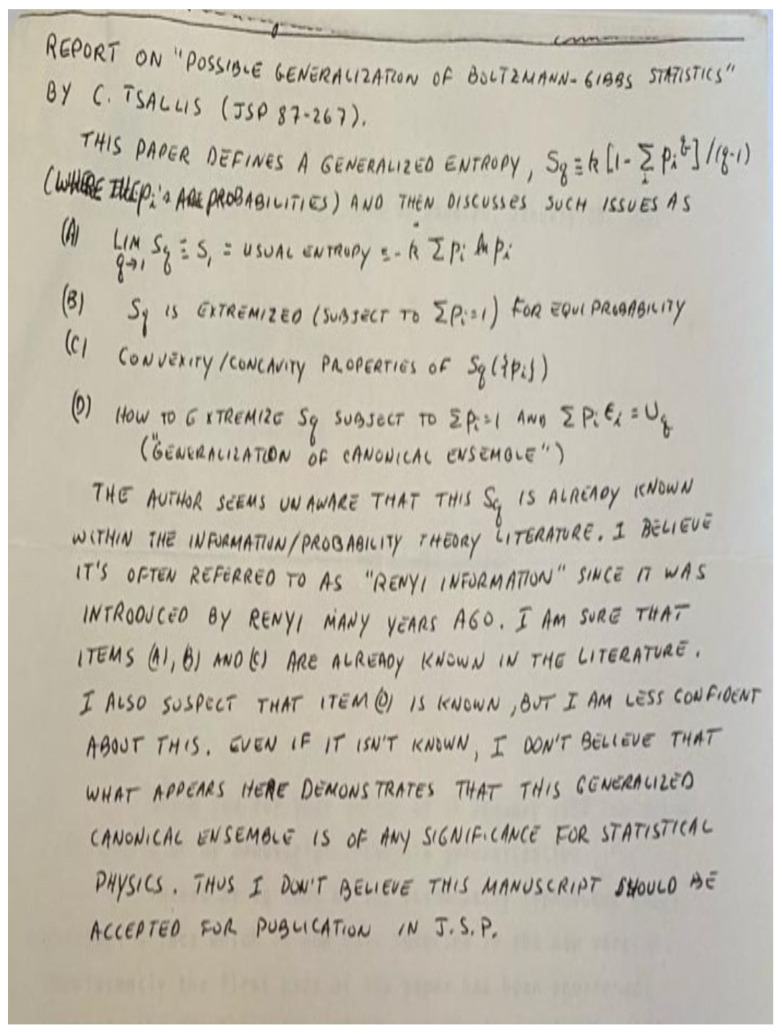
Report of the second reviewer of [11].

**Figure 4:**
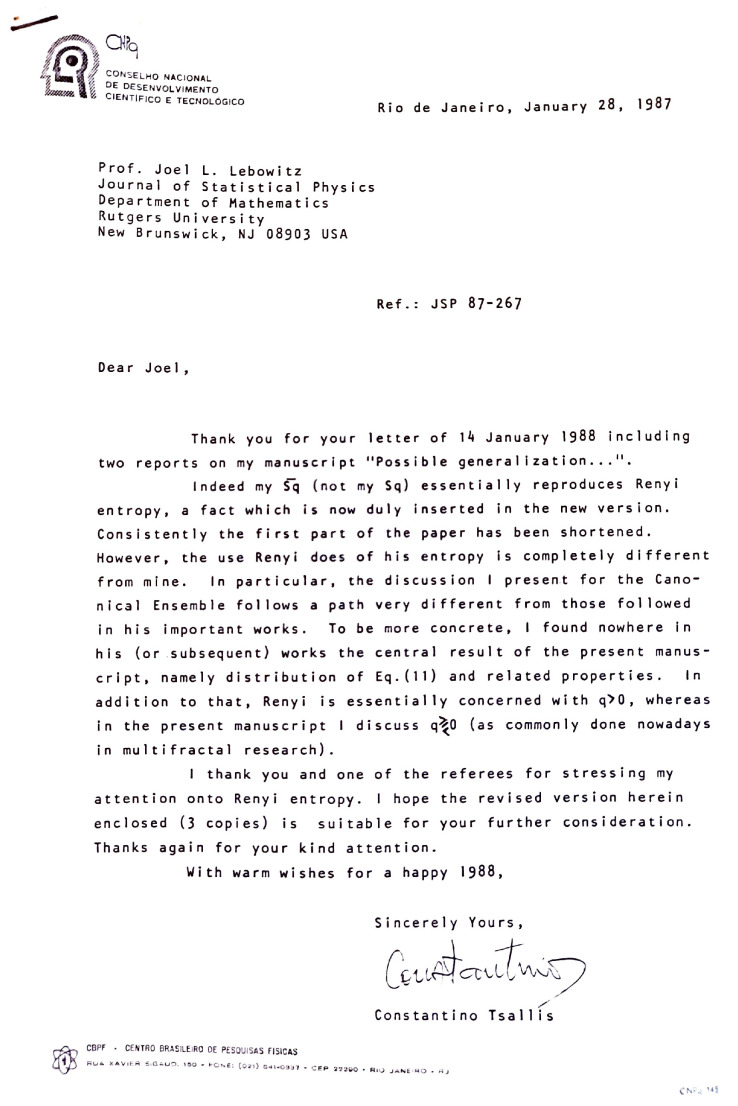
Submission of my revised version (the correct date is 28 January 1988, not 1987).

I first wrote a Centro Brasileiro de Pesquisas Físicas preprint (*Notas de Física CBPF-NF-062/87*), published in 1987. Eventually, I submitted the manuscript to the *Journal of Statistical Physics* [11] (this paper is, at the date I write these lines, the most cited one in the entire life of the journal, born in 1969, with 7164 citations at the Web of Science (All Databases), the second one, with 2715 citations, being the well-known 1978 article by Mitchell J. Feigenbaum, in **19**, 25–52), at the time edited by Joel L. Lebowitz. The submission letter, sent on my birthday, 5 November 1987, included the lines “Well, the last few months I have been working in a ’crazy’ idea: a possible generalization of Boltzmann–Gibbs statistics! […]. I did not succeed in finding a direct and useful application to an already known system. Nevertheless, the generalization has—at least to my eyes!—an internal elegance, which, I think, makes it worthy to be published: maybe somebody else will find the desired applications!”. As far as I can tell, the manuscript was sent to two reviewers and neither of them showed any enthusiasm. The first reviewer wrote “Although the ideas of this paper are not tremendously new, I recommend publication in the Journal of Statistical Physics”. The second reviewer appreciated the content of the paper even less. The report is here reproduced in Figure 3. It seemingly confuses $S_{q}$ with Rényi’s entropic functional ${\bar{S}}_{q}$ (independently rediscovered in Equation (8) of the 1988 paper), and ends with “I don’t believe that what appears here demonstrates that this generalized canonical ensemble is of any significance for statistical physics. Thus I don’t believe this manuscript should be accepted for publication in J. S. P.”. The editor, Lebowitz, sent to me these two reports on 14 January 1988. He wrote “My suggestion would be that you certainly include references to the Renyi entropy. I really do not know any offhand, but I certainly have seen it in the literature. I particularly remember seeing it mentioned in papers by Hao Bai-lin, but I believe that you will have any trouble finding it. Given that, you may want to shorten the paper and emphasize point D. I will be happy to go along with publication after I get your revised and shortened version”. I submitted my revised version on 28 January 1988 (misprinted as 1987): see Figure 4. The editor, Lebowitz, formally accepted its publication on 15 March 1988 and it was published in July 1988 (the differences between the original and the revised versions are the following ones: (1) after further discussions, “I am very indebted to E.M.F. Curado and H.J. Herrmann for very stimulating discussions” (in the 1987 version) became “I am very indebted to E.M.F. Curado, H.J. Herrmann, R. Maynard and A. Coniglio for very stimulating discussions” (in the 1988 version); (2) for reasons that are not present in my memory any more and in surprising contrast with what I practice in virtually all my publications, “We postulate for the entropy…” (in the 1987 version) became “I postulate for the entropy…” (in the 1988 version); (3) after learning of the existence of the Rényi entropy, I added (in the 1988 version) “For arbitrary *q*, ${\bar{S}}_{q}$ reproduces the Renyi entropy. ^(2)^” and consistently added the reference 2. A. Rényi, *Probability Theory*, (North Holland, 1970)”), three years after I started thinking about such a generalization of the celebrated centennial BG theory.

A few months later I was scheduled to deliver an invited talk at the *International Workshop on Fractals* organized by Luciano Pietronero and held during 10–15 October 1988 in Erice, Italy. Renowned scientists were also present, such as Michael E. Fisher, Benoit Mandelbrot and Shlomo Alexander, among others. I asked Pietronero whether I could talk for a few minutes on a topic that was not in my initial Abstract. He told me to feel free to use my time as I preferred. I then briefly presented the content of my 1988 paper during the last 10 min of my talk. Mandelbrot was ostensively showing his disapproval by negatively moving his head just in front of me. At the coffee break I approached him and gave a reprint of my article which had just arrived in my hands, while telling him “This is what I talked about”. He took the reprint and, in front of me, went directly to the references to check whether his name was there. As he did not find it, he gave me, on the spot, the reprint back and told me “*Tout ceci a été fait il y a bien longtemps*” (all this has been carried out a long time ago). It can be trivially checked that his discouraging statement was completely gratuitous. At the end of the meeting there was a collective appreciation of what the audience had liked the most. Hans J. Herrmann said “The last 10 min of Constantino”.

On 18 November 1988, S. Alexander sent to me from the University of California, Los Angeles, a long letter with his opinion on my Erice presentation. He included therein: “You presented it as a new path in statistical physics—in my view without any justification. My guess would be that the chances that this will prove useful are about equal to those of other attempts to violate the second law of thermodynamics—or generalize it. If I prove wrong I will concede that this is the greatest contribution to physics since Einstein”.

The next relevant step of *q*-statistics was performed thanks to Evaldo M.F. Curado: the article [24] established the first connection with thermodynamics through a *q*-generalized partition function and consistently generalized the third axiom of the celebrated Shannon theorem. (A rather funny incident occurred with this paper. We had used in it the word “holistic” in a sort of intuition that this theory, one day maybe, could be useful for globally correlated systems, a well-established fact nowadays. One of the reviewers strongly criticized the inclusion of that word (which was “esoteric” in his/her understanding) in a scientific paper. We did not agree with him/her, especially because in Greek this word is simply a sort of antonym of “atomistic”. But, to avoid an irrelevant controversy, we eliminated the word from our revised text. It was with amused surprise that we discovered, several months later, that the word reappeared in the published version, most probably due to some mild inadvertence at the production level of the journal!)

Then, in 1993, a long-awaited result appeared. Angel R. Plastino and his father, A. Plastino, published [25] the first application to a physical system, namely the stellar polytropes, introduced by Lord Kelvin in 1862. It was since long known that the extremization of the entropic functional $S_{BG}$ leads to a distribution which is characterized by an *infinite* total mass. This unphysical result disappears when it is $S_{q}$ which is extremized with *q* sensibly differing from unity. A. Plastino’s genuine interest in $S_{q}$ started during a long and relaxed conversation he and I had at the hotel swimming pool in San Juan, Puerto Rico, during the XVI International Workshop on Condensed-Matter Theories (1 to 5 June 1992). The Plastinos’ paper became accessible to me and Roger Maynard during the International Workshop on Nonlinear Phenomena, held during 7 to 9 December 1992 in Florianopolis, Brazil. During three or four hours, we peripatetically discussed it trying to understand why $S_{q}$ does the correct job where $S_{BG}$ fails. We concluded that it was due to the fact that gravitation is a *long-range* interaction. The door was open.

A couple of years later, on 4 April 1995, I delivered a talk at the Physics Department of the Boston University at the invitation of Harry Eugene Stanley. Bruce M. Boghosian was in the audience. Soon after, he produced a new bridge with a concrete physical system [26]: a non-neutral electronic plasma, where the Coulombian interactions play a role similar to the gravitational ones in stellar polytropes. Boghosian showed that the empirical distribution emerging in two-dimensional turbulence in a pure-electron plasma column precisely corresponds to q=1/2.

Concomitantly with the worldwide spread of scientific articles focusing on diverse aspects of *q*-statistics (at the date I am writing these lines, they surpass 10,000 articles authored by nearly 17,000 scientists from 112 countries, as they appear in the Bibliography at [27]), a wave of some opponents grew up around the world. Among them, it is possible to distinguish Joel L. Lebowitz—ironically enough, precisely the *Journal of Statistical Physics* Editor who accepted my 1988 paper for publishing, Itamar Procaccia—who, in March 2002, declared without any justification, to a Brazilian newspaper that all this was nothing but “mindless fitting”, Roger Balian—who was very fundamentally critical (Balian sent, nearly 25 years ago, a private letter to A.K. Rajagopal criticizing *q*-statistics and telling him that, in the 1978 Balian–Balazs paper, it was proved that basically no other statistical mechanics was possible outside the BG one; Rajagopal and Sumiyoshi Abe studied carefully the Balian–Balazs 48-page paper and then published, in [28], their mathematically consistent *q*-generalization of it; they naturally quoted the Balian–Balazs paper and sent to Balian their recently published paper together with a letter thanking his indication of the 1978 Balian–Balazs paper and its content; as far as I know, they never received an answer back from Balian) but declined the formal invitation from the President of the *Société Française de Physique* (SFP) to have, in Paris, a free public debate with me, organized by the SFP itself (some time after Balian’s declination, his own laboratory at L’Orme des Mérisiers, Commissariat à l’Énergie Atomique, France, invited me to deliver a seminar on 9 March 2009 on nonadditive entropies and the associated statistical mechanics; while the loudspeakers were announcing the beginning of the seminar, Balian was at his office, 5–6 m away from the seminar room, but he did not show; I cannot say that his attitude was a surprise to me; in contrast, the audience was quite interested and asking many questions, very especially Serge Aubry, who openly manifested his appreciation of the theory and its physical consequences), Michael Nauenberg—whom I invited, with all traveling and hosting expenses covered, to freely present his objections at the International Summer School and Workshop on “Complex Systems—Nonextensive Statistical Mechanics”, held during 30 July to 8 August 2006, in Trieste, Italy, sponsored by the International Centre for Theoretical Physics/ICTP Director Katepalli R. Sreenivasan (free time was given to Nauenberg to publicly present his viewpoints and criticism, and possibly hear some of the dozens of talks by all kinds of speakers focusing on *q*-statistics: he went to none), Peter Grassberger—who, both in private and publicly, confused $S_{q}$ with Renyi’s entropy in spite of the fact that the intervals of *q* for which these two entropic functionals are concave considerably differ, and a few others. With quite rare exceptions, such claims are not accompanied by concrete technical papers, which could in principle be answered/rebutted through other technical papers. This hardly constitutes a surprise: opinative claims are always by far easier than rigorously founded ones. This is but the old Greek distinction between *doxa* and *episteme*!

A contrasting and interesting case is that of Joseph I. Kapusta. On 19 May 2021, Kapusta delivered an online talk within the Theoretical Physics Colloquium series that Igor Shovkovy was hosting at the Arizona State University (ASU), USA. His talk was titled “A Primer on Tsallis Statistics for Nuclear and Particle Physics”. It started with a pedagogical introductory attempt and ended with skeptical comments about the *q*-generalized statistical mechanics being useful for discussing physical phenomena. I discovered, on the internet, the existence of Kapusta’s talk many months after it was delivered. I had plenty of reasons to disagree with him, and therefore I suggested to Shovkovy that he organized at ASU a in-person or online open debate with Kapusta, or at least a seminar by myself focusing on the points that Kapusta had criticized in his seminar. Shovkovy showed no special interest in organizing such (reciprocal) activity, so I decided to rebut, on general grounds, Kapusta’s views in an online seminar of mine at the Santa Fe Institute, New Mexico, which was delivered on 12 April 2022 [29]. The whole issue is focused on in an article of mine titled *Enthusiasm and skepticism: Two pillars of science—A nonextensive statistics case* [30].

**Figure 5:**
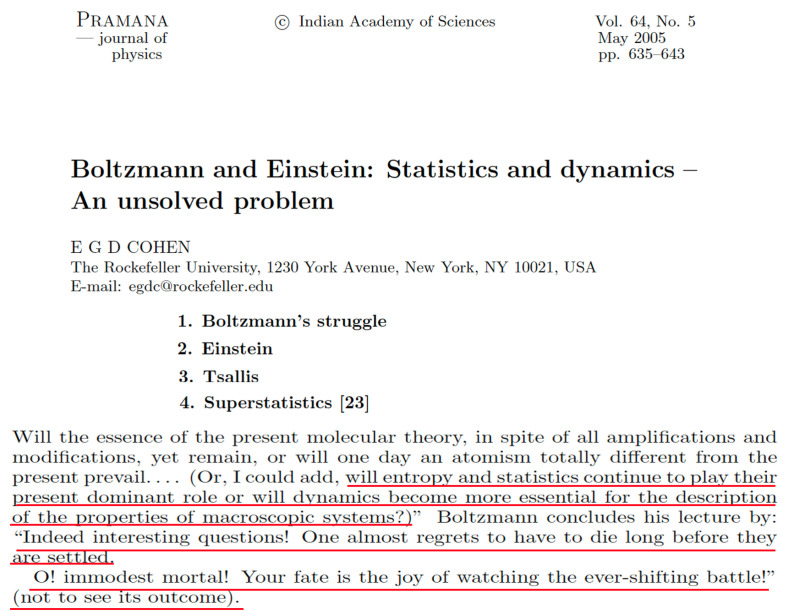
Basic content of Ezequiel G. D. “Eddie” Cohen’s Boltzmann Medal reception lecture in Statphys 22/IUPAP, held during 4 to 9 July 2004 in Bangalore, India. The red underlines are mine.

As it happens, we may also identify scientists who have expressed diametrically opposite opinions. These include Murray Gell-Mann—1969 Nobel laureate, who, after hearing my talk at the IIIrd Gordon Research Conference on “Modern Developments in Thermodynamics”, 18 to 23 April 1999 in Il Ciocco-Barga, Italy, stood up from his seat and came to the front of the auditorium exclaiming “Wonderful, absolutely wonderful!”, Pierre-Gilles de Gennes—1991 Nobel laureate, who, after a 40 min conversation during the International Conference on “Scaling Concepts and Complex Fluids”, 4 to 8 July 1994 in Catanzaro, Italy, shared with me that “a nonadditive entropy seems to me quite natural for gravitational systems.”, László Tisza—who kindly signed for me a copy of his book “Generalized Thermodynamics” [31] with the words “With best wishes to Constantino Tsallis for his bold enterprise to generalize Generalized Thermodynamics on a broad front. Laszlo Tisza. 8 April 1995” (I was introduced to László Tisza—highly esteemed, by the way, by Murray Gell-Mann, who considered him a top master in thermodynamics—by Gene Stanley, who invited him to his Boston University office in 1995 to introduce us to each other; on that occasion, Tisza was nearly 90 years old and we had a lengthy and delightful conversation; at the end, Tisza told me “How cute! I stopped working in statistical mechanics because I did not know which way to go. And, certainly, I never thought about generalizing entropy.”), Leo P. Kadanoff—who, after hearing the seminar that I delivered on 4 May 2005 at the University of Chicago at his invitation, told me, while going for dinner, “Everything that you said seems to me quite natural and not controversial at all.”, Athanassios S. Fokas—who, at the end of the talk that, through his invitation, I delivered on 15 November 2012 at the Department of Applied Mathematics and Theoretical Physics, Cambridge University, England, loudly exclaimed “Unbelievable, unbelievable!”, Ezequiel G. D. “Eddie” Cohen—who emphatically included *q*-statistics in his Boltzmann Medal reception lecture in Statphys 22/IUPAP, during 4 to 9 July 2004 in Bangalore, India [32], see Figure 5, George Contopoulos—who, on two different occasions, invited me to become a full member of the Academy of Athens; since I could not accept this tempting position because I would be unable to stay long enough per year in Greece due to my family in Rio de Janeiro, he eventually honored me, as President of the Academy of Athens, with its highest distinction, namely the *Aristion* (Excellence), Michel J. L. Baranger—who, during a workshop at the New England Complex Systems Institute in the 1990s, after we lengthily discussed nonlinear dynamical consequences of $S_{q}$, told me “I learnt something about physics today. It does not happen often to me.” [33], Bruce B. Boghosian—who, walking around within MIT, told me “General Relativity became possible through Riemannian geometry, which violates Euclid’s 5th postulate. You generalized the BG theory by violating the additivity of the usual entropic functional. It is but a neat illustration of Kuhn’s *Structure of Scientific Revolutions*” [34], Peter T. Landsberg—who once told me, walking around the São Conrado beach in Rio de Janeiro, “In the first pages of all books on thermodynamics it should be written that the content is valid only for short-range interactions, but it is not” and, smiling, he added “In mine it is.”, Roger Maynard, Christian Beck, Hans J. Haubold, J. Doyne Farmer, Shun’ichi Amari, Alan R. Bishop, H. Eugene “Gene” Stanley, Thomas A. “Tom” Kaplan, Grzegorz Wilk, Tamás S. Biró, Gergely G. Barnaföldi, Jan Naudts, Stefan Thurner, Rudolf Hanel, Andrea Rapisarda, Alessandro Pluchino, Alberto Robledo, Renio S. Mendes, Ugur Tirnakli, Sabir Umarov, Giorgio Benedek, Guiomar Ruiz, Antonio Rodriguez, Piergiulio Tempesta and definitively many others.

The whole situation might be accurately described through the acute Gregoire Nicolis and David Daems’ words [35] *“It is the strange privilege of statistical mechanics to stimulate and nourish passionate discussions related to its foundations, particularly in connection with irreversibility. Ever since the time of Boltzmann it has been customary to see the scientific community vacillating between extreme, mutually contradicting positions."*.

**Figure 6:**
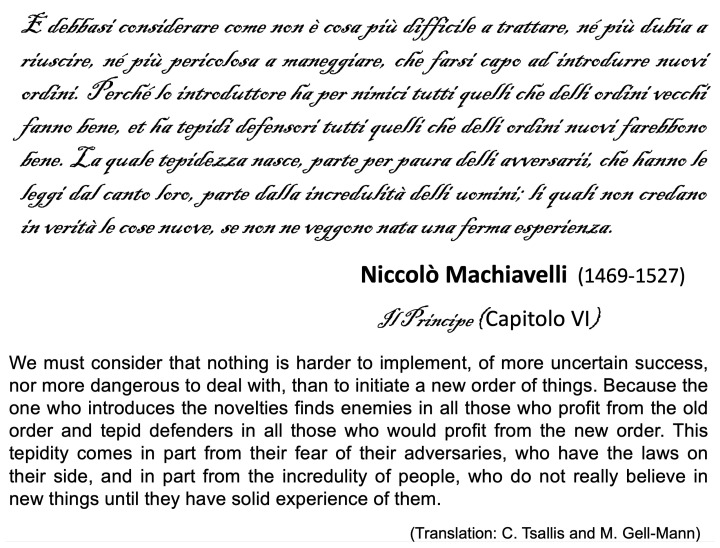
Text from Nicolas Machiavelli (translated into English by M. Gell-Mann and myself during a pleasant afternoon at the Santa Fe Institute, New Mexico).

It could even be described through Niccolò Machiavelli’s words: see Figure 6.

## 4. Perspectives

**Figure 7:**
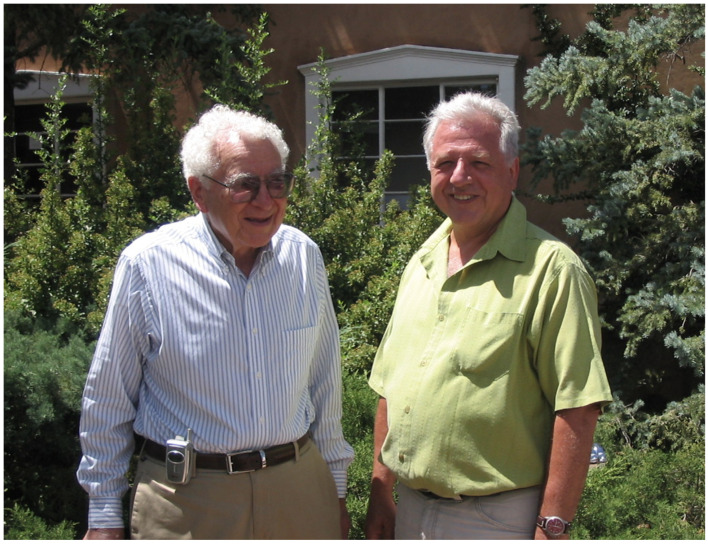
With M. Gell-Mann at the Santa Fe Institute, New Mexico (2005).

I was chatting one day at tea time with Gell-Mann at the Santa Fe Institute (see Figure 7) and he shared with me that he was traveling to California to deliver a talk on the *Laws of creativity*. I inquired: *Oh, how interesting! Tell me about these laws.* He continued: *The first of them is: If you have good reasons to believe in something, you must believe in all of its consequences, no matter how strange or foolish these consequences might a priori seem to you! If you believe that the molecules of the air of this room are in Brownian motion, you must believe that they are all the time hitting in the cheeks of your face as well!* I would say that this “law” is true more generally than just for physical phenomena. Reviewers of my 1988 manuscript were skeptical about the validity or usefulness of a nonadditive entropy in physics. After four decades of all kinds of applications, and of experimental and analytical validations in both inanimate and living matter (my friend and distinguished chemist Ricardo de Carvalho Ferreira, whose name is for ever linked to the asteroid 158520 (2002 FR1), generously told me once “You did for living matter what Boltzmann did for inanimate matter”; a similar view was kindly expressed to me by Aneta Stefanovska during the Medyfinol 2012 meeting in Santiago de Chile; she then invited me to publish a paper in Contemporary Physics, which was indeed implemented [36]; living matter frequently involves complex stationary or quasi-stationary states; what is currently referred to as *thermal equilibrium*, quite frequent in inanimate matter, may be seen as a particular case of stationary state; see also [37]), it is definitively allowed to think that those JSP reviewers were wrong, and so were, at least in their initial thoughts, Mandelbrot and Alexander and various others. At variance, Herrmann, Curado, Plastino Sr. and Plastino Jr., Boghosian, Beck, Gell-Mann, de Gennes, Tisza, Cohen, Landsberg, Kadanoff, Maynard, Haubold, Tirnakli, Nobre, Borges, Deppman and many others, were right!

After all, $S_{BG}\left(A+B\right)$ is symmetric (with regard to A↔B) and, for independent *A* and *B*, it is *additive* [38] in $S_{BG}\left(A\right)$ and $S_{BG}\left(B\right)$. $S_{q}$ is symmetric and *multilinear* (the importance of a strategic multilinearity property was emphasized to me, four or five decades ago, by Carlos Guido Bollini during a quick chat at CBPF) in $S_{q}\left(A\right)$ and $S_{q}\left(B\right)$ (trivial consequences of the specific $p_{i}^{q}$-dependence postulated in definition (6)). Just a “small” logical step further! But so amazingly powerful!

This is perhaps not the appropriate place for registering the many (impressive) validations of the present thermostatistical theory that are available in the literature: they can be found in [20,39,40,41], for instance. But it might be appropriate to mention at this point a few illustrative ongoing issues.

(i) Long-range-interacting many-body Hamiltonian systems undoubtedly need to be revisited. Let us focus on *d*-dimensional classical systems with two-body attractive power-law interactions whose potential decays with distance *r* like $1/r^{\alpha }$, where α≥0. I initially thought, for many years, that 0≤α/d≤1 required q≠1, whereas systems with α/d>1 were fully correctly described within BG statistical mechanics. This belief was based on the elementary fact that α/d>1 potentials are integrable. But increasing evidence is growing nowadays that this integrability is *necessary but not sufficient* for the BG theory to be applicable. It appears that *all* momenta of $1/r^{\alpha }$ need to be *finite* and not only the lower-order ones. Consequently, only when α/d→∞ should we use q=1 if we wish that *all* thermostatistical (e.g., energy and velocity distributions) and nonlinear dynamical (e.g., size dependence of the maximal Lyapunov exponent) properties are adequately handled within the BG theory. Consistently, q=1 is also fully correct if the two-body interaction potential decays exponentially with distance or if it is nonzero only between near-neighboring bodies. For the power-law case, the energy distribution may be reasonably conjectured to be given by a *q*-exponential form (the *q*-exponential function is defined as $e_{q}\equiv {\left[1+\left(1-q\right)x\right]}_{+}^{\frac{1}{1-q}}$ with ${\left[\cdots \right]}_{+}=\left[\cdots \right]$ if [⋯]>0 and zero otherwise; $e_{1}^{x}=e^{x}$; its inverse function is given by $\ln_{q}x\equiv \frac{x^{1-q}-1}{1-q}$; $\ln_{1}x=\ln x$) with *q* given by say $q=\frac{4}{3}$ for 0≤α/d≤1 and $q=1+\frac{1}{3}e^{1-\alpha /d}$ for α/d>1. In such power-law systems, only the α/d→∞ limit is to be considered, as mentioned above, as rigorously *short-range* interactions, belonging therefore to the BG world.

Moreover, it would be wonderful to (analytically and/or numerically) check whether, for α/d∈[0,∞), a nonuniform convergence occurs at the t→∞ and N→∞ limits, something like the existence, in the (N,t) space, of a curve (probably of the type $1/N\propto {\left(1/t\right)}^{\gamma }$, with γ>0) such that on one side ($1/t^{\gamma }\ll 1/N$) BG statistics prevails, whereas on the other side ($1/t^{\gamma }\gg 1/N$) *q*-statistics prevails (see, for instance, [42]).

The rigorous approach of this class of systems would provide an analytical confirmation that Boltzmann–Gibbs statistical mechanics is *sufficient but not necessary* for the validity of (properly scaled) thermodynamics and its Legendre-transform structure (see [43,44] as well as pioneering studies such as [45,46] and references therein).

(ii) For the astonishing quantum chemical reaction studied in [47], it has been conjectured [48] that the index *q* associated with the distribution of velocities nearly satisfies $\left(q-1\right)\propto n^{1/4}$, where *n* is the $H_{2}$ density. Further experimental work would be very welcome to check the possible validity of this conjecture.

(iii) A “dream” theorem [49] is waiting to be proved, namely, what would be the necessary and sufficient conditions for a *q*-generalized Central Limit Theorem whose attractors (in the space of probability distributions) would be *q*-Gaussians instead of the usual Gaussians. Both of them are ubiquitously found in nature.

(iv) The elegant *q*-generalization of the product frequently referred to, in the literature, as the *q-product* [50,51] has been recently shown to be consistent with an entire new *q*-generalized *algebra* [52,53]. Could some sort of *q*-generalized *vector space* be defined on this basis? Such an achievement could be of great operational utility in areas such as theoretical chemistry where *q*-Gaussians are known to play a sensibly more efficient numerical role than Gaussians, as Kleber C. Mundim has repeatedly shown.

**Figure 8:**
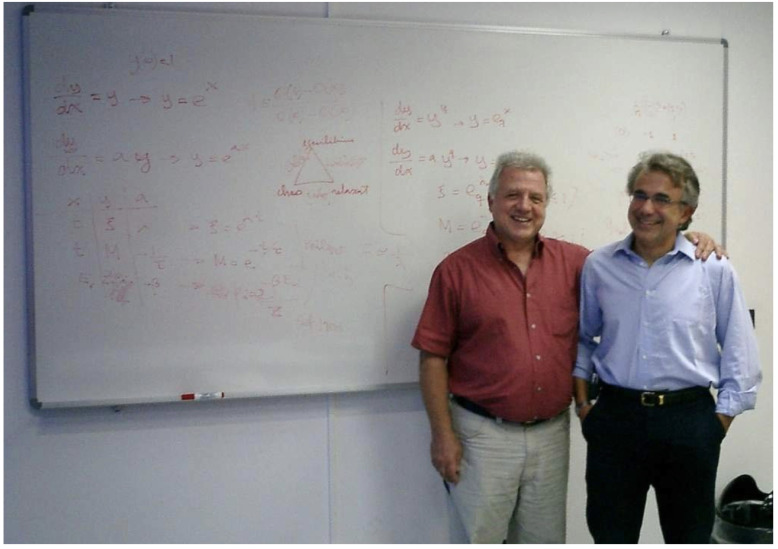
Seminar in Catania, 2003, with Andrea Rapisarda. On the blackboard we see the proposal of the *q*-triplet, profusely found afterwards in nature and nonlinear dynamical systems. Such a possibility was based on possible physical interpretations of the ordinary differential equation $dy/dx=ay^{\;q}$ [y(0)=1 yields $y=e_{q}^{\;a\;x}$], thought up in May 1988 on the train from Bayreuth back to Copenhagen (where I was visiting Per Bak at the Niels Bohr Institutet) to provide an analytical basis for the re-association in folded proteins [55]. It was George Bemski who drew my attention to this peculiar biophysical phenomenon, telling me that it could well be related to *q*-statistics: he was right!

(v) Triangles and more triangles! Andrea Rapisarda invited me in 2003 to deliver a seminar at the Dipartimento di Fisica e Astronomia of the Università di Catania, Italy. At the end of the presentation, a student expressed his curiosity with regard to the fact that I had mentioned different values of *q* for the same physical system. I clarified that, for a complex system, it was possible that different physical properties behave *q*-exponentially *with different values of q*, whereas a system well described within BG statistical mechanics currently exhibits only one value of *q*, namely q=1. I then illustrated that with a triplet, more precisely $\left(q_{sen},q_{rel},q_{stat}\right)$ (*sen*, *rel* and *stat* stand for *sensitivity*, *relaxation* and *stationary state*, respectively): the *q*-triplet was born. See Figure 8 and [54].

**Figure 9:**
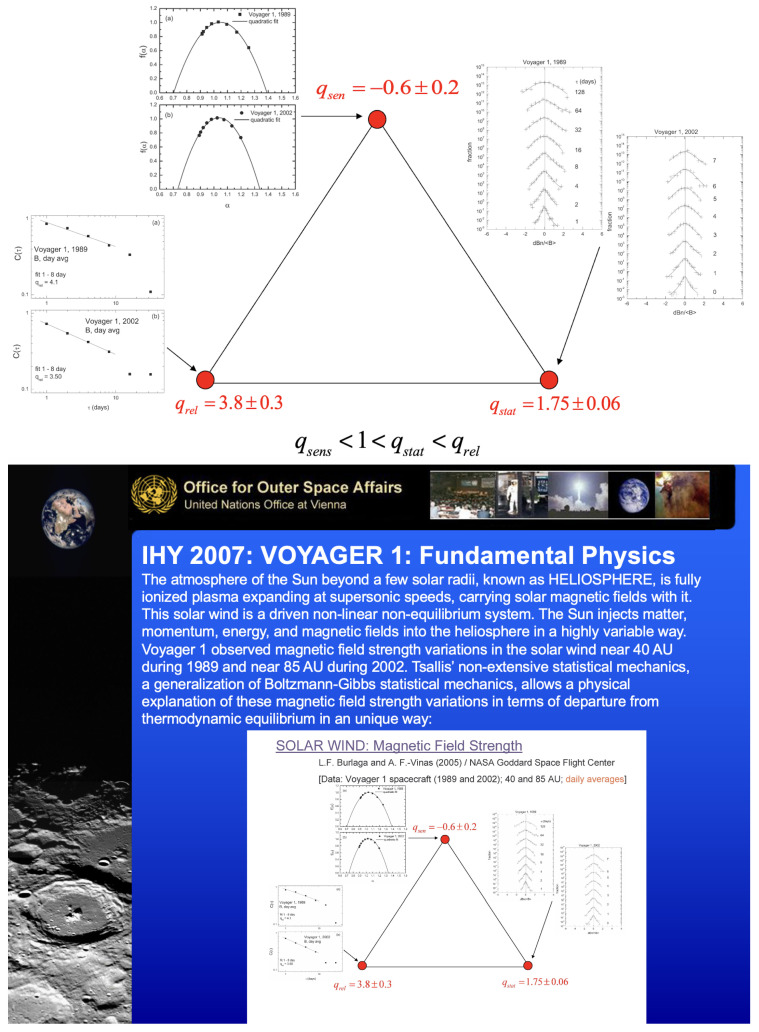
**Top**: Slide exhibiting the NASA results on the solar wind *q*-triplet ($q_{sensitivity}<1<q_{stationary\;state}<q_{relaxation}$ was the expectation in [54]). **Bottom**: With my consent for using the slide, this poster was prepared, selected and exhibited by the United Nations Office for Outer Space Affairs for the Opening Ceremony of the United Nations International Heliophysical Year exhibit (19 February 2007, Vienna).

**Figure 10:**
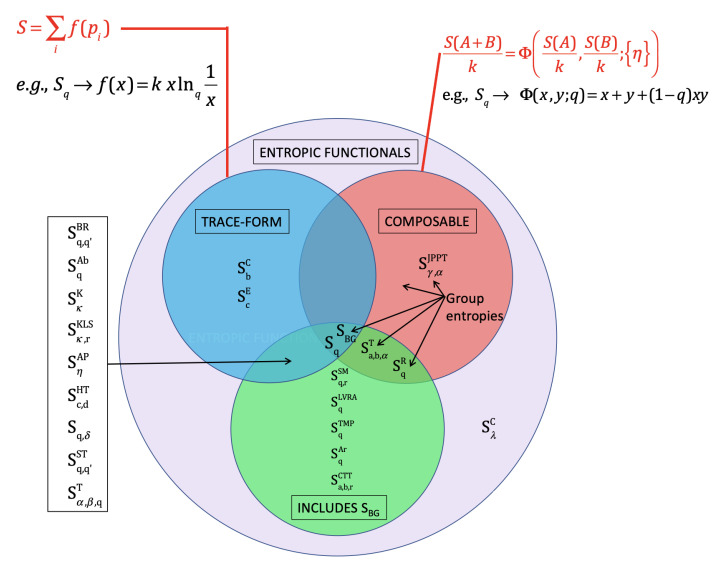
Scheme of the Enciso–Tempesta theorem [61] proving the uniqueness of $S_{q}$ for simultaneously being trace-form and composable, and containing $S_{BG}$ as a particular case. For further details, see [20].

**Figure 11:**
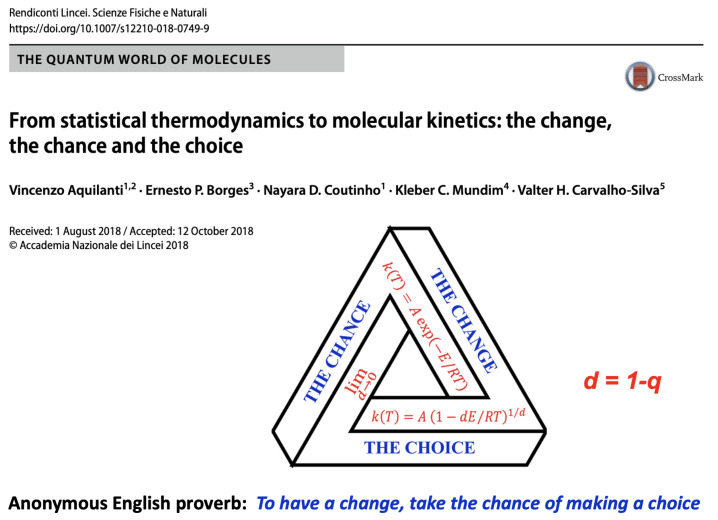
Triangle from [68], reflecting the *q*-exponential generalization of the Arrhenius law for chemical kinetics. Experimental validations of q≠1 can be found in [68] and references therein.

**Figure 12:**
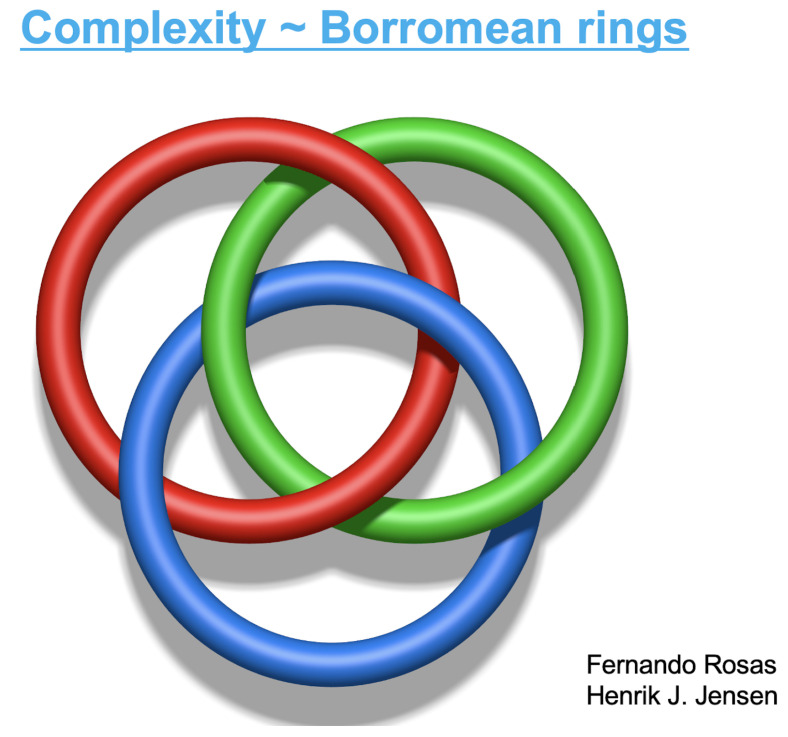
Borromean rings are three elementary rings that are two-by-two free but not so when the three of them are entangled. This expressive metaphor for complex systems was recently presented in a lecture by Henrik J. Jensen, in co-authorship with Fernando Rosas.

A couple of years later, in January 2005, NASA invited me to deliver some talks at the Goddard Space Flight Center, Greenbelt, Maryland. It was terribly cold but the warm hospitality of Leonard F. Burlaga and Adolfo Figueroa Viñas balanced that! Len Burlaga showed to me the clock where data from the Voyager 1 spacecraft were arriving. To see directly online those numbers sent to the Earth from near Pluto meant for me an unforgettable experience. Then, in his office, Len showed to me corresponding time series of the solar wind. It struck me that these data could perhaps reveal the empirical existence in nature of the *q*-triplet conjectured in Catania. He asked me how to obtain these three numbers from his Voyager 1 data. I explained to him with all details how this might possibly be carried out. A few weeks later I received at the Santa Fe Institute-SFI, New Mexico, where I was spending a long sabbatical period, an email that Len sent me with wonderful news: $\left(q_{sen},q_{rel},q_{stat}\right)=\left(-0.6\pm 0.2,3.8\pm 0.3,1.75\pm 0.06\right)$. See Figure 9 and [56]. The first *q*-triplet ever detected in nature was found, amazingly enough, in the solar wind! I immediately gave a seminar at SFI. Murray Gell-Mann became intrigued with these numbers arriving from outer space and came to my office on a Friday afternoon, just before the party at which his 75th birthday was going to be celebrated. Through a several-hour discussion, we succeeded in finding simple relations between those three numbers, based on the *additive duality* q↔(2−q) and the *multiplicative duality* q↔1/q. The theoretical proposal was $\left(q_{sen},q_{rel},q_{stat}\right)=\left(-1/2,4,7/4\right)$. That was the beginning of an entire algebra of indices *q*! [20,57]. I asked Murray why he was so fond of triangles. His answer was “You ask *me* why I like triangles? To start with, it is the simplest possible polygon!” From that moment on, a plethora of *q*-triplets started being observed around the world in very diverse complex systems. I conjecturally proposed some possible logical frame to those empirical sets of *q*-triplets and advanced a connection with the Moebius group of transformations in [58,59], but the real step forward [60] was made by Jean-Pierre Gazeau, my teaching colleague at the University of Paris, close to 55 years ago! Consistently with that first triangular structure, all kinds of triangles emerged within *q*-statistics. An important one is indicated in Figure 10, which illustrates the Enciso–Tempesta theorem [61], proving that the only entropic functional which simultaneously is trace-form and composable, and contains the BG entropy as a particular case is $S_{q}$ as given in Equation (6). In the realm of nonlinear dynamical systems, we may think of the three classical roads to chaos (period doubling, quasi-periodicity and intermittency) as one more triplet deeply related to *q*-statistics, as profusely shown by Robledo, Tirnakli, Beck, Jensen and their collaborators (see [62,63,64,65,66,67] and references therein), among others. Another interesting triangle emerged in [68] in connection with molecular kinetics as shown in Figure 11. Finally, a beautiful metaphor for complex systems was recently presented by Henrik J. Jensen at the IIIrd International Workshop on Statistical Physics, held from 13 to 15 December 2023 in Antofagasta, Chile: see Figure 12.

*On top* (or, rather, *at the basis*) of all the above, let us remind the reader of a distinguished and crucial triplet upon which nonextensive statistical mechanics is constructed. We refer to the behavior of the *q*-exponential function $e_{q}^{-x}$, which straightforwardly emerges through the optimization of $S_{q}$ under simple constraints. Indeed, $e_{q}^{-x}$ decays exponentially for q=1, as an asymptotic power-law $x^{-\frac{1}{q-1}}$ for q>1, and presents a cutoff for q<1.

(vi) A *nonadditive* entropic functional differing from $S_{q}$, namely

(7) $$S_{\delta }=k\sum_{i=1}^{W}p_{i}{\left[\ln\left(1/p_{i}\right)\right]}^{\delta }\;\;\;\left(\delta >0;S_{1}=S_{BG}\right)\;,$$

was introduced in [69,70], which, *for equal probabilities*, satisfies $S_{\delta }=k{\left(\ln W\right)}^{\delta }$. The quantum version of $S_{\delta }$ was advanced in [20,71] as a *thermodynamically admissible* alternative to the Bekenstein–Hawking entropy $S_{1}$ for black holes as well as for cosmological holographic models. Indeed, for such deeply gravitational systems (*if* thought of as (3+1)-dimensional ones), $S_{1}$ is well known to be proportional to the black hole area *A* and not to its volume. Therefore, $S_{1}$ is *not* extensive and violates, consequently, the Legendre structure of thermodynamics. (Two decades ago, during a garden cocktail in a scientific event in Germany, Antonio Coniglio asked me “Since you have generalized the entropy, why don’t you generalize the Legendre structure of thermodynamics itself?”. His provocative question haunted my thoughts for many years, until I became deeply convinced that the specific form of the entropic functional acts on an epistemological level *less fundamental* than the elegant and powerful Legendre-transform structure of macroscopic phenomena. Unless new deep and solid empirical evidence emerges, this structure can (and should), through proper and natural scalings, be maintained in theoretical physics as it stands today, *even if the entropic functional differs from the usual BG one*. The basic dilemma for complex systems is whether to keep the *additive* entropic functional $S_{BG}$ and violate the entropic extensivity mandated by the thermodynamical Legendre structure, or the other way around. It turns out eventually that violating the usual entropic additivity is a small price to pay in order to preserve the important Legendre structure. The situation is totally analogous to the special relativity dilemma of preserving the Galilean additivity of composition of velocities and violating the Lorentz transformation, or the other way around. It was clear to Einstein that violating the lovely Galilean additivity was a small price to pay for preserving the Lorentz transformation, which enabled nothing less than the unification of Maxwell electromagnetism and mechanics!) It was claimed in [71] that $S_{\delta }$ with δ=3/2 could solve the serious thermodynamical difficulty of $S_{1}$. (The idea of using $S_{\delta }$ for black holes emerged at the closed International Symposium on “Sub-nuclear Physics: Past, Present and Future” organized by Antonino Zichichi at the Pontifical Academy of Sciences during 30 October to 2 November 2011. After my presentation and that of Michael J. Duff, we had an interesting coffee-break conversation focusing on the thermodynamical requirement of entropic extensivity for all macroscopic systems, mandated by the Legendre structure of thermodynamics. Motivated by our discussion, I went back to my room at Saint Martha’s House inside the Vatican and started investigating which value of q≠1 could possibly make $S_{q}$ overcome the inadmissible lack of extensivity of the well-known Bekenstein–Hawking entropy (which corresponds to q=1). It took me two research evenings to suddenly realize that perhaps no such value of *q* did exist: I had to use an entropic functional different from $S_{q}$! I then remembered about $S_{\delta }$, which I had introduced, as a mere mathematical possibility, in [69]. The path was open and eventually led to δ=3/2 for (3+1)-dimensional black holes.) Basically, if lnW∝A, then $S_{\delta =3/2}\propto A^{3/2}$, which, as desired, is *extensive*. Recent observational results are accumulating [72,73,74] which indeed indicate δ≃3/2. (Ref. [72] indicates δ=1.565 for neutrinos as detected at the IceCube Neutrino Observatory at the South Pole. Ref. [73] indicates δ=1.87 and δ=1.26 through two different theoretical processings of the data collected at the outer-space Planck Observatory/ESA; amazingly enough, the mean value of 1.87 and 1.26 precisely yields δ=1.565! Ref. [74] indicates, from the Big Bang nucleosynthesis and the relic abundance of cold dark matter particles, δ=1.499.) This appears to neatly exclude, for such systems, the Bekenstein–Hawking entropy $S_{1}$ (i.e., δ=1), *enfant aimé* of string theorists and others (see, for example, [75]). The scientific importance of such timely issue surely deserves a re-analysis in the light of *nonadditive entropic functionals* adequately chosen so as to satisfy *entropic extensivity*.

Human memory is like a fractal, one reminiscence endlessly pulling another one, and another and another. Still, I hope that, through these lines, I could share with the reader a few illustrative facets of what it is possible to learn and appreciate during half a century of theoretical physics.

In the present manuscript, I mainly focused on concepts—*truth and beauty*—that may be thought of as primarily belonging to what is currently referred to as the *objective* world. There are others, equally important, such as *curiosity and enthusiasm* (from Greek *enthousiasmós*, divine inspiration), *focus and resilience*, which primarily belong to the *subjective* world…but that is another story!

At this point, as final words, it might be proper to emphasize that, in science and elsewhere, the concepts of *objective* and *subjective* themselves surely are strangely entangled. In the prologue of Miguel de Unamuno’s wonderful *Niebla* (1935) we can read: *Don Quijote me ha revelado íntimos secretos suyos que no reveló a Cervantes.* (Don Quixote revealed to me intimate secrets of himself that he did not reveal to Cervantes).
